# Wavelet-based protocols for ion channel electrophysiology

**DOI:** 10.1186/2046-1682-6-3

**Published:** 2013-03-14

**Authors:** Armin Kargol

**Affiliations:** 1 Physics Department, Loyola University New Orleans, New Orleans, LA, 70118, USA

**Keywords:** Voltage-gated Shaker potassium channel, Markov model, Wavelet, Patch clamping

## Abstract

**Background:**

Fluctuation-induced phenomena caused by both random and deterministic stimuli have been previously studied in a variety of contexts. They are based on the interplay between the spectro-temporal patterns of the signal and the kinetics of the system it is applied to. The aim of this study was to develop a method for designing fluctuating inputs into nonlinear system which would elicit the most desired system output and to implement the method to studies of ion channels.

**Results:**

We describe an algorithm based on constructing the input as a superposition of wavelets and optimizing it according to a selected cost functional. The algorithm is applied to ion channel electrophysiology where the input is the fluctuating voltage delivered through a patch-clamp experimental apparatus and the output is the whole-cell ionic current. The algorithm is optimized to aid selection of Markov models of the gating kinetics of the voltage-gated Shaker K^+^ channel and tested by comparison of numerically obtained ionic currents predicted by different models with experimental data obtained from the Shaker K^+^ channels. Other applications and optimization criteria are also suggested.

**Conclusion:**

The method described in this paper can be useful in development and testing of models of ion channel gating kinetics, developing voltage inputs that optimize certain nonequilibrium phenomena in ion channels, such as the kinetic focusing, and potentially has applications to other fields.

## Background

Ion channels in cellular membranes are proteins that form gated pores to allow passive transport of ions down their electrochemical potential gradient [1]. They open and close in response to an appropriate gating stimulus, such as transmembrane voltage, ligand binding, or mechanical stress. In this paper we concentrate on voltage-gated ion channels. Their gating can be mathematically described as a Markov chain with voltage-dependent transition rates between a small set of discrete states. Such a model corresponds to a microscopic picture of the channel as a macromolecular assembly undergoing conformational changes. The gating is a generalized motion in a certain “energy landscape” and the discrete Markov states correspond to local energy minima. The transition rates reflect thermally-activated jumps over energy barriers separating the minima. In practice, both the topology (the number and connectivity of the discrete states) as well as the transition parameters of such models are developed to fit the experimental data that comes mostly in a form of electrophysiological recordings [2-9].

The technique of patch-clamping in ion channel electrophysiology allows measurements of currents flowing through the channels in cellular membranes [10]. When a glass micropipette containing a recording electrode touches the cell membrane it forms a tight seal (a gigaseal) around a patch of the membrane. Thus clamped patch can be ruptured, creating a direct electrical connection with the cytoplasm and allowing measurements of ionic currents through the entire cell surface (the whole-cell mode), or maintained. In the latter case the recording is of currents flowing through this small patch only, which contains very few, or even just a single channel (the single-channel mode). Typical recordings are performed under the voltage-clamp conditions, where the membrane potential is controlled by a patch-clamp amplifier and the resultant current measured. A specific form of the applied voltage depends on the details of the channel gating kinetics that the protocol is intended to probe. For instance, the activation protocols are based on a voltage step up from a hyperpolarized value, at which channels are closed, to various depolarized values. These protocols test the activation of channels at different voltages. In contrast, the tail protocols are designed to observe channel closing (deactivation) due to a re-polarizing voltage step. The channels are subjected to a depolarizing prepulse of a certain duration, the result of which is the opening of a number of channels. It is then followed by a re-polarizing voltage step and the channel deactivation (closing) can be observed.

These two protocols illustrate the current paradigm on which a vast majority of electrophysiological experiments are based: the voltage protocols are piecewise constant and consist of only few voltage steps at discrete times [2-9]. From a physical point of view this corresponds to observing an ensemble of ion channels under equilibrium or near-equilibrium conditions. For a constant voltage, the distribution of channels among discrete Markov states reaches an equilibrium form. Following a voltage step, this equilibrium is disturbed, but the ensemble simply relaxes to a new equilibrium distribution corresponding to the new voltage value. An entirely different approach, where the voltage fluctuates on a time scale comparable to the relaxation times of the channel kinetics has been discussed [10-17]. This approach forces the channels into nonequilibrium distributions which may lead to new phenomena not observable under equilibrium conditions. These types of fluctuation-induced effects in nonlinear systems are well known in various other areas of physics and in recent years there has been a growing interest in investigating such phenomena in biological systems, including ion channels. One of the most studied examples is the stochastic resonance [17-22], where a noise, either intrinsic or added to the system, improves the system response to weak time-dependent signals. Other examples include ratchets [23,24], resonant activation [25-28], or the nonequilibrium kinetic focusing [29-31].

Several of these effects can be very promising in investigating and controlling the kinetics of ion channels. For instance, one of the main goals of ion channel research is to develop a model of channel gating kinetics. Most commonly used are the discrete Markov chains, however even the basic features of these models are still disputed, e.g. cooperativity or Markovian character of gating. It has been suggested [10,11,16,17] that this ambiguity results partially from a very incomplete set of experimental data and by expanding the data we can develop better and more unique models. In essence, if we have several models that adequately reproduce the existing set of experimental data, these models can be tested and disproved by comparing to new experimental data until one or more models fail to match them. One of the ways the available data sets can be expanded is by using fluctuating voltages in voltage-clamp electrophysiological experiments, contrary to a currently preferred method of applying piecewise constant voltages. The problem with this approach is in deciding what type of fluctuating voltage input would be most useful. In the previous studies the dichotomous noise has been used [10,11] but the exact properties of the noise (amplitude, frequency, temporal asymmetry) have been chosen arbitrarily. What is needed is a systematic method of selecting a fluctuating voltage input that would generate maximally different responses from channel models, thus allowing us to efficiently select the model most compatible with all existing experimental data.

A related issue is that of controlling the gating of ion channels. It has an enormous practical importance in biology and medicine. So far the dominant approach is to use pharmacological agents (drugs and toxins) or global electric fields (defibrillator shocks) to modify channel behavior. A much more subtle approach, aimed at forcing ion channels into a specific conformational state has been also proposed [29]. Known as the nonequilibrium kinetic focusing, the method is based on applying fluctuating voltage to ion channels in order to enhance transitions into a selected state and suppress transitions out of that state. As a result, in the ensemble of ion channels, i.e. in a cell, most channels will occupy a selected state. This is also a nonequilibrium effect and is not achievable in standard electrophysiology protocols. The kinetic focusing has been studied numerically and analytically [29,31] using the dichotomous noise stimulation but it is entirely unclear what type of stimulation would be optimal to achieve the maximum focusing in a desired state. These are two examples of problems in ion channel research where one needs a systematic method of selecting a fluctuating voltage for use in patch-clamping experiments which would optimize the experimental output, either by producing maximally divergent responses from kinetic models of gating, thus facilitating model selection, or by focusing ion channels into a specific conformational state.

In this paper we describe in detail such a method of designing an input to a system that maximizes a desired output. It is based on constructing a fluctuating signal as a superposition of wavelets in a dyadic wavelet basis and optimizing the wavelet coefficients for a specific system response. We developed the method for use with the experimental technique of patch-clamping but it could be applied in a variety of contexts where fluctuating system inputs are used. We concentrate in this paper on applications to ion channel electrophysiology, in particular since our method represents a radical departure from the current paradigm. We show how one can synthesize a signal with desired properties and implement it in a voltage-clamp experiment with ion channels.

We illustrate the method by applying to one of the two problems mentioned earlier in the introduction, i.e. by developing voltage protocols that maximize the difference between various Markov models for the same ion channels. The numerical results are compared to experimental data obtained from Shaker K^+^ channels. The idea of this method was mentioned in our previous work on the nonequilibrium response spectroscopy technique [17]. A version of this method was also used to analyze ionic currents in human heart sodium channels [16]. A related method, where a pulse composed of wavelets is designed to maximize a response of nonlinear systems has been also proposed recently in engineering [32].

## Methods

### Markov models of ion channels

**Figure 1:**
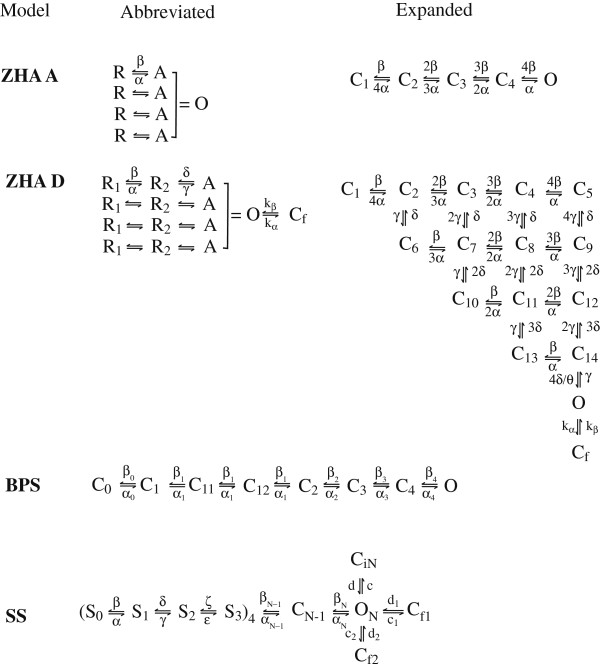
**Examples of Markov models for Shaker IR K**^**+ **^**channels.**

Ion channel gating results from rearrangements of the tertiary structure of the channel proteins, i.e. transitions between certain meta-stable conformational states of the molecule, in response to changes in trans-membrane potential. These molecular states can be either conducting (open – O) or non-conducting (closed – C or inactivated – I). Examples of Markov models for the Shaker K^+^ channels are shown in Figure 1. Transitions between the states are thermally activated and, in case of voltage-gated channels, are voltage-dependent according to [33]:

(1) $$\alpha \left(V\right)=\alpha \left(0\right)\exp\left(\mathrm{qV}/\mathrm{kT}\right)$$

where *α* is a generic transition rate, *V* – is the membrane voltage, *k* – the Boltzmann constant, and *T* – the absolute temperature. *q* is the so-called gating charge characterizing the molecule’s sensitivity to external electric fields. Such a description is only a coarse approximation and more precisely the channel gating should be viewed as a motion of a “gating particle” in a certain energy landscape, subject to thermal fluctuations and governed by the Langevin or Fokker-Planck equation [29,31,34-36]. However, a picture of channel gating as a discrete Markov chain with the discrete states corresponding to the minima in this energy landscape has been very successful. It disregards the internal structure of each energy well, but there is very little evidence that this structure has any significant effect on macroscopically observable quantities in most applications. In practice, the main task in functional ion channel studies is to determine the topology of Markov models (i.e. the number, type and connectivity of discrete states) as well as the parameters for the state transition rates (the gating charges *q* and the rates at 0 mV membrane potential). The model topology and model parameters are determined by fitting model responses to various sets of experimental data. As we argued in the past [17], the data used for model development is usually incomplete, since it consists mostly of responses to piecewise-constant voltage stimulation. The result is that several distinct models can be developed for the same kinetic data.

### Wavelet decomposition of voltage inputs

The purpose of this paper is to describe and implement a method of constructing fluctuating voltage inputs for ion channel electrophysiology to obtain a certain type of response from ion channels. The method exploits the interplay between spectro-temporal patterns of the signal and the gating kinetics of the channel molecules. It requires simultaneous control over temporal and spectral properties of the voltage pulse. A tool that offers this kind of time-frequency localization is the wavelet analysis, developed in the last two decades [37,38]. Wavelets are “localized waves”, i.e. oscillating functions *ψ* concentrated around a certain region in the argument space with zero mean:

(2) $$\int_{-\infty }^{\infty }\psi \left(t\right)\mathrm{dt}=0$$

and finite “energy”:

(3) $$E=\int_{-\infty }^{\infty }{\left|\psi \left(t\right)\right|}^{2}\mathrm{dt}<\infty$$

There are many different families of wavelets, some with compact support. In essence, the wavelet analysis is similar to Fourier analysis, however it has certain advantages. In Fourier transform the testing functions (trigonometric) have infinite support hence they are not very well suited to analysis of nonstationary signals. Fourier transform provides spectral information about the signal, however temporal information, although not lost, is hidden in the phases of the sines and cosines. By proper translation and dilation of the testing wavelet, the wavelet transform can provide both spectral and temporal information, i.e. it shows not only what frequencies are present, but also when they are present in a nonstationary signal.

Wavelet analysis begins with a mother wavelet, i.e. a function satisfying conditions (2)-(3). The simplest is the Haar wavelet:

(4) $$\psi \left(t\right)=\left\{\begin{array}{ll} 1 & 0\leq t<\frac{1}{2}\\ -1 & \frac{1}{2}\leq t<1\\ 0 & \mathrm{elsewhere} \end{array}\right)$$

which is also the first member of the Daubechies family of wavelets. By varying the dilation parameter *a* and the translation parameter *b* we obtain from the mother wavelet a set of normalized wavelets of the form:

(5) $$\psi_{a,b}\left(t\right)=\frac{1}{\sqrt{a}}\psi \left(\frac{t-b}{a}\right)$$

The continuous wavelet transform CWT is the convolution of the signal *x(t)* with the members of this wavelet set:

(6) $$T\left(a,b\right)=\frac{1}{\sqrt{a}}\int_{-\infty }^{\infty }x\left(t\right)\psi_{a,b}\left(t\right)\mathrm{dt}$$

The CWT coefficients *T(a,b)* can be plotted versus the dilation and translation parameters (*a* and *b*). More common are plots of the wavelet energy density *E(a,b) = |T(a,b)|*^*2*^, known as the scalograms. The coefficients *T(a,b)* (or the energy *E(a,b)*) carry the information about how much the signal is compatible with the wavelet at scale *a*, at location *b*. One may also notice that since the wavelet is a function with oscillation within a certain, relatively narrow, frequency range, then moving to a different scale amounts to changing the dominant frequency of the wavelet. This can be expressed in terms of the mean wavelet frequency *f*_*0*_[38]. In that sense a scalogram shows spectro-temporal patterns in the signal.

In principle, the CWT is an expansion of a signal *x(t)* into an uncountable set of wavelets {*ψ*_*a,b*_}. Some wavelets, such as the Daubechies family, have been designed so that a countable subset of dilated and translated wavelets, of the form:

(7) $$\psi_{m,n}=2^{-m/2}\psi \left(2^{-m}t-n\right)$$

constitutes an orthonormal basis in *L*^2^(ℜ). In other words, every signal *x(t)* with finite “energy”, defined as

(8) $$E=\int_{-\infty }^{\infty }{\left|x\left(t\right)\right|}^{2}\mathrm{dt}$$

can be approximated arbitrarily well by a finite superposition of basis elements *ψ*_*m,n*_. Such a basis is called the dyadic wavelet basis. Using wavelets (7) we can define the discrete wavelet transform (DWT) as:

(9) $$T_{m,n}=2^{-m/2}\int_{-\infty }^{\infty }x\left(t\right)\psi_{m,n}\left(t\right)\mathrm{dt}$$

Just like for the CWT, the DWT coefficients can be plotted versus the scale and translation indices (*m* and *n*), yielding the discrete transform plot. Since on the axes one has discrete indices, the plot has a block form. Knowing the DWT coefficients, a signal can be synthesized using:

(10) $$x\left(t\right)=\sum_{m,n}T_{m,n}\psi_{m,n}\left(t\right)$$

One of the applications is the so-called multiresolution analysis (MRA). It is an iterative procedure where a given signal is separated into a coarse approximation and the detail. The latter is expressed as a superposition of wavelets at a certain level *m* (with a given scale parameter *m*), while the former is expressed in terms of the companion function – the scaling function. Next the level-*m* approximation is separated into a coarser, level-*m + 1* approximation and the detail. The latter is again a superposition of wavelets at scale *m + 1*. Ultimately, the signal can be written as a sum of the scaling function and a combination of wavelets at different scales. The orthogonality of wavelets assures that the signal details at different scales are uncorrelated. The DWT given by equation (9) is discrete in scale and position, but still is a continuous function of time. In practice signals are discretely sampled. In this case in each iteration of the MRA both the approximation and the details are subsampled by a factor of 2. The process can continue until the “coarsest” level (i.e. the mean value) is reached.

The technique we use to construct signals (voltage inputs) with desired properties is based on the inverse DWT (10) and is essentially the opposite of the MRA we just described. For a chosen wavelet type (we tested the Haar wavelets since they most closely resemble currently used voltage step protocols and the Daubechies 8 wavelets for their compact support and the degree of smoothness) we synthesized the signal using (10) and including a finite number of levels. This number of MRA levels was determined by physiological considerations and the bandwidth of the recording apparatus. The latter was typically of the order of 5–10 kHz, and from previous studies [10-17] we expected the effect of the fluctuating voltages to be concentrated in the 1–2 kHz region, hence we considered voltage waveforms composed of 8–10 MRA levels. Rather than imposing a finite energy requirement in the form (8) we put a constraint on the amplitude of the voltage fluctuations not to exceed a physiologically reasonable value. We chose 200 mV peak-to-peak since larger oscillations resulted in a quick loss of a gigaseal.

### Channel model response and input optimization

In the previous paragraph we described how a signal with desired properties can be synthesized in a dyadic wavelet basis. We should also specify what these “desired” properties are. The voltage input is selected to elicit a certain response from ion channels. The method we are describing in this paper is flexible enough for a variety of purposes. It can be used to synthesize inputs that maximize differences in computed ionic currents between various models, hence aiding model development and testing, or inputs that maximize the parametric sensitivity of the model, as described in [13]. Another possible application to ion channels would be to test for the phenomenon called the nonequilibrium kinetic focusing, described in a numerical studies of ion channels [29,31], and recently investigated experimentally [30]. The technique can be also used in applications to other fields [32].

In order to determine the model output, let us consider an *n*-state Markov model for an ion channel (see e.g. Figure 1). The current state of the channel can be described as an *n—*component normalized vector ***P****(t)* of probabilities *{P*_*i*_*(t)}* of finding a channel in various Markov states. For a large ensemble of channels, e.g. in whole-cell mode patch-clamping experiments, these equal the state occupancies. The time evolution of the system is described by the master equation:

(11) $$\frac{dP\left(t\right)}{\mathrm{dt}}=W\left[V\left(t\right)\right]P\left(t\right)$$

where the ***W****[V(t)]* is the transition matrix consisting of the transition rates (1) between various states. For a variable voltage input a formal solution to (11) can be written in a form:

(12) $$P\left(t\right)=\exp\left\{\int_{0}^{t}W\left[V\left(0\right)\right]\mathrm{ds}\right\}P\left(0\right)$$

where ***P****(0)* is the equilibrium probability distribution at the holding potential. We compute (12) numerically by iteration:

(13) $$P\left(t_{i+1}\right)=\exp\left(Wt_{i}\right)P\left(t_{i}\right)$$

with a sufficiently small time step. ***P****(t)* represents the time evolution of the probability distribution. If the goal is to get a model output that is comparable with the experimentally measurable quantities, such as ionic currents, then the model current can be computed from the probability distribution using the Ohm’s law:

(14) $$i\left(t\right)=g_{0}g\left(V\right)\left(V-V_{r}\right)O\cdot P\left(t\right)$$

Here *g*_*0*_*g(V)****O·P****(t)* is the total conductance, where ***O·P****(t)* is the projection of the probability vector onto the open state(s), *g(V) -* the nonlinear, voltage dependent part of the conductance, and *g*_*0*_ is the overall scaling factor dependent on the number of channels in the ensemble and the conductance of an open channel.

### Genetic algorithm

The algorithm for voltage input synthesis is an optimization procedure. Depending on the goals we define the “cost functional” for the model output and then modify the inputs to optimize this functional. We use a version of a genetic algorithm applied in other contexts previously [10-17]. We begin with a first generation voltage pulse obtained from (10) with a set of randomly chosen wavelet coefficients *T*_*m,n*_. From this set of coefficients we obtain a number, typically 10–20, of second generation sets by random perturbations according to:

(15) $$T_{\mathrm{daughter}}=T_{\mathrm{parent}}\left(1+E_{0}\exp\left(-\mathrm{Rn}\right)\left(X-0.5\right)\right)$$

where *T* is one of the wavelet coefficients, *n* numbers the generations of the algorithm, *E*_*0*_ and *R* are convergence parameters characterizing the initial range of the random perturbations (*E*_*0*_) and the rate at which the search narrows down with each generation (*R*), and *X* is a uniformly distributed random number from the interval (0,1). In practice *X* was generated using the pseudorandom number generator in Matlab, and for a run of 15,000 generations we used *E*_*0*_ = 0.5 and *R* = 0.0004. For each of the daughter sets we synthesize the input according to (10), compute the model output using (13), and evaluate the cost functional. The daughter set that optimizes the cost functional is chosen as the parent for the following generation. The procedure is iterated but as eq. (15) shows at each generation the range of random perturbations used to generate the daughter sets decreases. There are different possible variations of this scheme, where for instance the input is optimized level by level or all levels at once. Other methods are also possible for constructing the new daughter sets, for instance in [32] a differential evolution is described where the new generation inputs are obtained not by multiplicative random perturbation of the chosen “parent”, but according to a simple additive formula involving the “parent” and several randomly chosen intermediate sets. As mentioned in the previous section, there are practical constraints on this optimization procedure. For instance, the maximum amplitude of voltage fluctuations must be restricted to a certain range.

### Channel electrophysiology

As an illustration of this numerical scheme we constructed voltage inputs designed to maximize the difference in model outputs between different Markov models for the Shaker K^+^ channels shown in Figure 1. The simulated data were compared to experimental currents recorded from the channels stably expressed in tSA201 cells. The details of experimental design were described previously [12-14]. Our electrophysiology apparatus consists of the Axopatch 200B amplifier with a CV 203 BU headstage (Axon Instruments Inc., Union City, CA), Nikon TS100 inverted microscope Nikon Instruments Inc., Lewisville, TX), Sutter MP-285 micromanipulator (Sutter Instrument Co., Novato, CA). The experiments were performed at 12°C, maintained by Physitemp temperature controller (Physitemp Instruments Inc., Clifton, NJ). Fluctuating voltage inputs were prepared as binary files with Matlab (Mathworks Inc., Natick, MA), read into the Pulse program (HEKA Electronik Gmbh, Lambrecht, Germany) and converted to analog form by ITC18 AD/DA converter (Instrutech Corp., Great Neck, NJ). The ionic currents were digitized at a rate between 50 and 200 kHz and stored on a hard disk. All analysis was performed with custom programs written in Matlab.

We studied a mutant Shaker channel (Shaker K Sk1), stably expressed in tsA201 cells (gift from D. Hanck). The cells were cultured in 35 mm Corning Petri dishes (Corning) in DMEM medium (ATCC, Manassas, VA) supplemented with 10% FBS (ATCC, Manassas, VA), 1% penicillin/streptomycin (Gibco BRL, Gaithersburg, MD) and 200 μg/ml Zeocin (Invitrogen, Carlsbad, CA) at 37°C in 5% CO_2_ in a CO_2_ incubator (Fisher Scientific, Pittsburgh, PA). Patch clamping pipettes were pulled from borosilicate capillary glass (Warner Instruments LLC, Hamden, CT) on a Sutter pipette puller (Sutter Instrument Co., Novato, CA).

## Results

### Models for Shaker K^+^ channels

As an illustration of the proposed method we consider one of the main goals in ion channel studies – the development of kinetic models of channel gating. Such models are developed by fitting to various types of experimental data (mostly various ionic current recordings). If the set of experimental data is small, the number of models compatible with it is typically very large. Model refinement is achieved by introducing new types of data (e.g. gating currents) and by increasing the number of different voltage protocols. The paradigm of electrophysiology is to use voltage protocols consisting of static voltages changing only stepwise at few discrete times. Typical examples are the activation and tail protocols. Very few studies used randomly fluctuating voltages such as the dichotomous noise [10-12].

Several models have been developed for the Shaker K^+^ channels. We consider here the Bezanilla-Peroso-Stefani (BPS) [3], Schoppa-Sigworth (SS) [6], and the Zagotta-Hoshi-Aldrich (ZHA) [9] models (Figure 1). The BPS model is a simple linear chain of 8 states and was developed based on the activation and deactivation (tail) protocols, as well as “on” and “off” gating currents. The model contains 5 independent pairs of transition rates (forward *α* and backward *β*). According to (1) there are two model parameters (the rates at 0 mV and the gating charges) per transition. Zagotta et al. proposed several models in a series of 3 papers [7-9] describing a number of experiments based on stepped voltage protocols. The ZHA A and D models, which we analyze here, also take into account basic knowledge of the channel structure. Namely, it is believed that the Shaker channel consists of four symmetrical subunits, each of which undergoes an activation process. It is mostly postulated that the subunit activation is independent from each other and the channel becomes open when all four subunits are activated. The ZHA A model is the simplest possible such topology. The four subunits can exist in two states only (resting R, and activated A) and the activation of all of them is equivalent to the opening of the channel. The transition rates between the R and A states are the same for all subunits. This topology can also be represented in its expanded form where states C_1_ to C_4_ describe the channel conformations with different number of subunits in the A state (0 for C_1_, 1 for C_2_, 2 for C_3_, 3 for C_4_). The conformation with all four subunits activated is the open (O) state of the channel. The transition rates in the expanded topology follow directly from the rates in the abbreviated version hence the model involves only 1 pair of transition rates (4 model parameters). The ZHA D model introduces two complications. First, the subunit kinetics now involves two resting states, so it is itself a linear chain of three states. Second, there is another closed state (C_f_) accessible only from the open state. Both corrections were introduced to improve the model fit to a variety of available experimental data. The expanded version of this topology is no longer linear and involves 16 distinct channel states, however the number of model parameters is the same as for the abbreviated version (three pairs of transition rates, i.e. 12 parameters). In that respect the BCS model is significantly more complex and computationally demanding, with 20 parameters. Finally, the SS model is the most complex [6]. Like the ZHA D model, it is rooted in the idea of 4 subunits undergoing a number (three in this case) independent transitions followed by two additional transitions that describe the concerted motion of the subunits. It also has three inactivated states reachable from the open state only. In the fully expanded form (not shown) this model has 38 states.

For all the models shown in Figure 1 we can write the transition matrices, according to (11). For instance the matrices for models ZHA A and BPS are as follows (for the ZHA D and SS models the matrices are 16 × 16 and 40 × 40, respectively, and are shown in the sample programs, see Additional files 1 and 2):

Model ZHA A:

(16) $$\left(\begin{array}{ccccc} -4\alpha & \beta & 0 & 0 & 0\\ 4\alpha & -\beta -3\alpha & 2\beta & 0 & 0\\ 0 & 3\alpha & -2\beta -2\alpha & 3\beta & 0\\ 0 & 0 & 2 & -3\beta -\alpha & 4\beta \\ 0 & 0 & 0 & & -4\beta \end{array}\right)$$

Model BPS:

(17) $$\left(\begin{array}{cccccccc} -\alpha_{0} & \beta_{0} & 0 & 0 & 0 & 0 & 0 & 0\\ \alpha_{0} & -\beta_{0}-\alpha_{1} & \beta_{1} & 0 & 0 & 0 & 0 & 0\\ 0 & \alpha_{1} & -\beta_{1}-\alpha_{1} & \beta_{1} & 0 & 0 & 0 & 0\\ 0 & 0 & \alpha_{1} & -\beta_{1}-\alpha_{1} & \beta_{1} & 0 & 0 & 0\\ 0 & 0 & 0 & \alpha_{1} & -\beta_{1}-\alpha_{2} & \beta_{2} & 0 & 0\\ 0 & 0 & 0 & 0 & \alpha_{2} & -\beta_{2}-\alpha_{3} & \beta_{3} & 0\\ 0 & 0 & 0 & 0 & 0 & \alpha_{3} & -\beta_{3}-\alpha_{4} & \beta_{4}\\ 0 & 0 & 0 & 0 & 0 & 0 & \alpha_{4} & -\beta_{4} \end{array}\right)$$

### Voltage protocols optimized for maximal divergence of model responses

**Figure 2:**
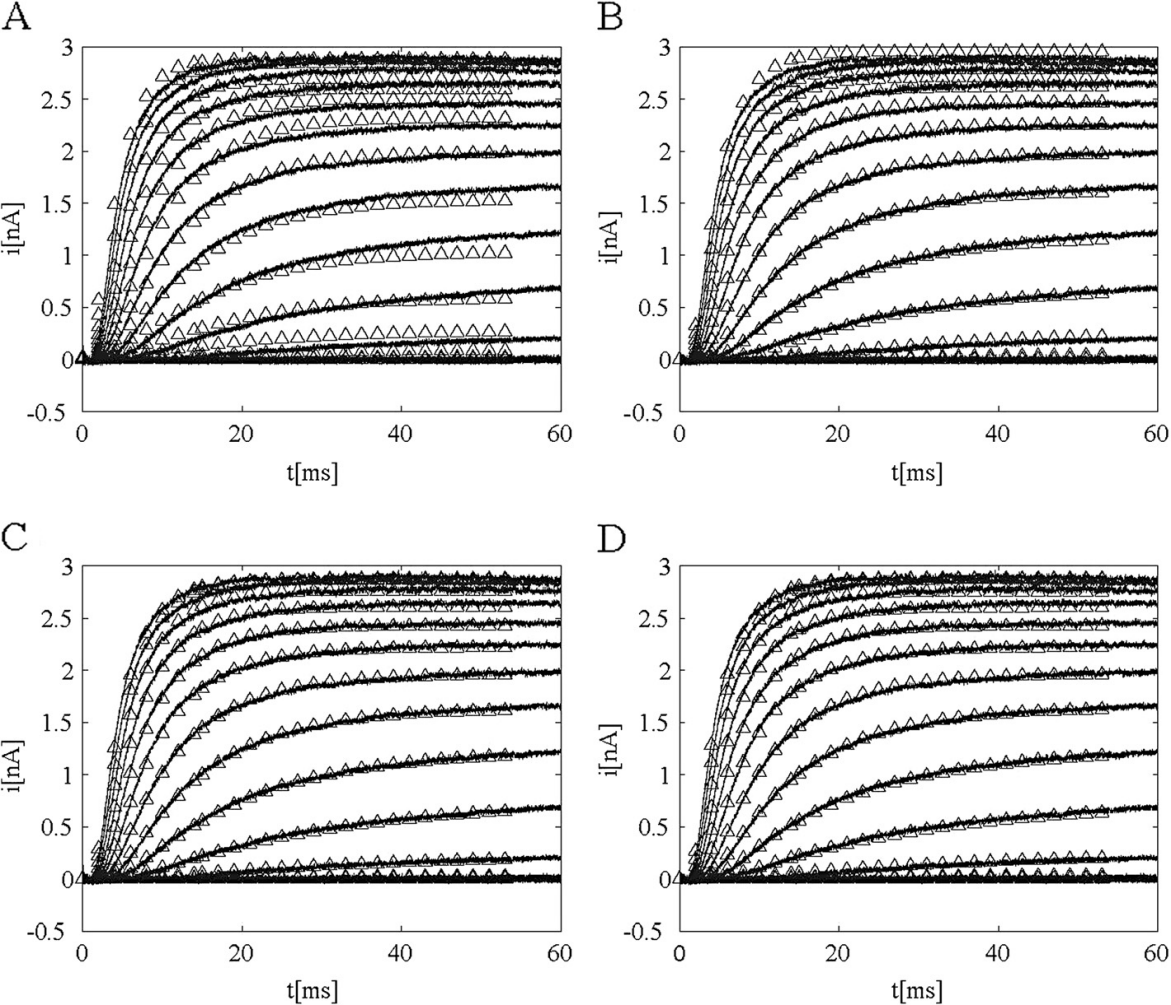
**Comparison of model currents (triangles) and experimental ionic currents (solid line) for activation protocols for four Markov models of Shaker K**^**+ **^**channels.** The voltage protocol consisted of holding potential of −90 mV followed by a series of steps to potentials from −70 mV to +42 mV in 8 mV increments. Data is shown for models: **A**) ZHA A, **B**) ZHA D, **C**) BPS, **D**) SS.

The primary criterion for a model selection is its ability to accurately reproduce various types of data. It is the choice of the type of data and the number of different data sets that determine how good and how unique the model is. The models described in the preceding sections were all developed using mostly different voltage-step protocols. As such they reproduce the experimental ionic current for any similar voltage-step protocol very well. Figure 2 shows model currents and the experimental current for the four models. Model parameters are listed in Table 1. Model ZHA A is a notable exception and it clearly fails even such simple test. Of the remaining three it is impossible to tell based on the typical activation and tail protocols which is the best approximation of the underlying molecular kinetics.

**Table 1:** **Model parameters used for current simulations using eq. (14)**

**Model**	**Rate amplitudes (ms**^**-1**^**)**	**Gating charges (units of e)**	**Additional parameters**
**ZHA A**	α(0) = 0.1219	q_α_ = 0.6232	$\begin{array}{ccc} g_{0} & = & 0.9717;g\left(V\right)=-1.9823\times 10^{-10}\times V^{5}-1.4013\times 10^{-9}\times V^{4}\\ & + & 1.0424\times 10^{-6}\times V^{3}-1.8204\times 10^{-5}\times V^{2}-1.3480\times 10^{-3}\times V\\ & + & 0.07696 \end{array}$
	β(0) = 0.0342	q_β_ = −0.0207	
**ZHA D**	α(0) = 2.1605	q_α_ = 1.9760	θ = 8.8660
	β(0) = 0.0558	q_β_ = −0.0108	$\begin{array}{ccc} g_{0} & = & 1.3011;g\left(V\right)=3.4123\times 10^{-11}\times V^{5}+6.8293\times 10^{-10}\\ & \times & V^{4}-7.6790\times 10^{-8}\times V^{3}-1.0646\times 10^{-8}\\ & \times & V^{2}-2.4698\times 10^{-4}\times V+0.04219 \end{array}$
	γ(0) = 0.2642	q_γ_ = 0.2080	
	δ(0) = 0.1103	q_δ_ = −0.5311	
	k_α_(0) = 0.3505	q_kα_ = 0.0138	
	k_β_(0) = 0.5253	q_kβ_ = −0.0203	
**BPS**	α_0_(0) = 0.2761	q_0_ = 0.7467	$\begin{array}{ccc} g_{0} & = & 0.9386;g\left(V\right)=-1.2009\times 10^{-11}\times V^{5}-1.5047\times 10^{-9}\times V^{4}\\ & + & 7.1616\times 10^{-8}\times V^{3}+2.3240\times 10^{-6}\times V^{2}\\ & - & 3.2463\times 10^{-4}\times V+0.03649 \end{array}$
	β_0_(0) = 1.2647	q_1_ = 1.4896	
	α_1_(0) = 1.6095	q_2_ = 0.7891	
	β_1_(0) = 0.1061	q_3_ = 0.8988	
	α_2_(0) = 1.6809	q_4_ = 0.8376	
	β_2_(0) = 0.2955	δ_0_ = 0.2082	
	α_3_(0) = 2.0483	δ_1_ = 0.1316	
	β_3_(0) = 0.8917	δ_2_ = 2.1390	
	α_4_(0) = 1.8216	δ_3_ = 0.3814	
	β_4_(0) = 0.3384	δ_4_ = 0.2731	
**SS**	α(0) = 1.0859	q_α_ = 0.8288	$\begin{array}{ccc} g_{0} & = & 0.9119;g\left(V\right)=-2.9371\times 10^{-11}\times V^{5}-3.6458\times 10^{-10}\times V^{4}\\ & + & 9.4630\times 10^{-8}\times V^{3}-2.0430\times 10^{-6}\times V^{2}\\ & - & 1.0250\times 10^{-4}\times V+0.03638 \end{array}$
	β(0) = 0.1415	q_β_ = −1.1574	
	γ(0) = 2.2983	q_γ_ = 0.0160	
	δ(0) = 1.3948	q_δ_ = −0.1893	
	ε(0) = 1.3100	q_ε_ = 0.0639	
	ζ(0) = 0.5676	q_ζ_ = −0.0584	
	α_N-1_(0) = 4.5890	q_αN-1_ = 0.0771	
	β_N-1_(0) = 0.1965	q_βN-1_ = −0.0779	
	α_N_(0) = 1.9438	q_αN_ = 0.6513	
	β_N_(0) = 0.1431	q_βN_ = −0.6569	
	c(0) = 0.0054	q_c_ = 0.0980	
	d(0) = 0.3853	q_d_ = 0	
	c_1_(0) = 0.1680	q_c1_ = 0	
	d_1_(0) = 3.9279	q_d1_ = −0.3678	
	c_2_(0) = 0.8233	q_c2_ = 0	
	d_2_(0) = 9.9927	q_d2_ = 0	

**Figure 3:**
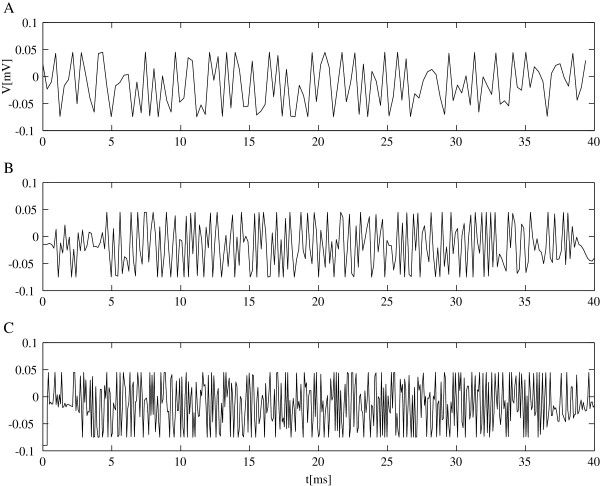
**Sample voltage protocols optimized for model current difference.** ZHA A vs. SS model with **A**) 8 wavelet levels, **B**) 9 wavelet levels, and **C**) 10 wavelet levels.

We used these models with the parameters optimized for the fit to standard activation and tail protocols. Our goal was to construct new fluctuating voltage waveforms that produce maximal differences in model responses (i.e. model currents) and thus could be used to select one of the models over the others. We used the numerical procedure described in Methods to construct the new voltage input as a superposition of wavelets. After computing the model response to this voltage input, i.e. the model ionic current, using eq. (14), we defined the difference in model response as the χ^2^ error between the corresponding model currents (see Additional file 3). The voltage input was then iteratively optimized through the genetic algorithm to maximize model divergence. We have done this for different pairings of the models (ZHA A vs. SS, ZHA D vs. BPS, etc.) and for different number of wavelet decomposition levels. A sample MATLAB code for ZHA D vs. SS is shown in Additional file 4. We used the Daubechies 8 wavelets with 8, 9, and 10 levels of MRA, which corresponded to the frequency range commensurate with the recording bandwidth of the patch-clamping apparatus. Figure 3 shows three samples of such voltage protocols with different number of wavelet levels, i.e. different frequency content.

### Comparison of model currents and experimental currents

Ionic currents were recorded from tSA201 cells stably transfected with the Shaker IR potassium channels, as described in Methods. We used the typical activation and tail protocols. The former were obtained for voltage steps from a holding potential of −90 mV to a series of values from −70 mV to 42 mV in 8 mV intervals. For the latter the protocol consisted of a 32 mV prepulse of 30 ms duration followed by a series of voltage steps from −120 mV to 48 mV in 12 mV intervals. The capacitative transients were removed from the experimental data using the standard P/4 method. The wavelet-based voltage pulses, described in the previous section, were implemented on our recording apparatus as described in the Methods. We monitored the bandwidth by obtaining the cell’s capacitance and the series resistance. Only cells with bandwidth exceeding 5 kHz were considered. For capacitative current transients we used the P/2 method, used also in our earlier paper [15], which has been effective in removing these transients even for continuously varying voltages.

**Figure 4:**
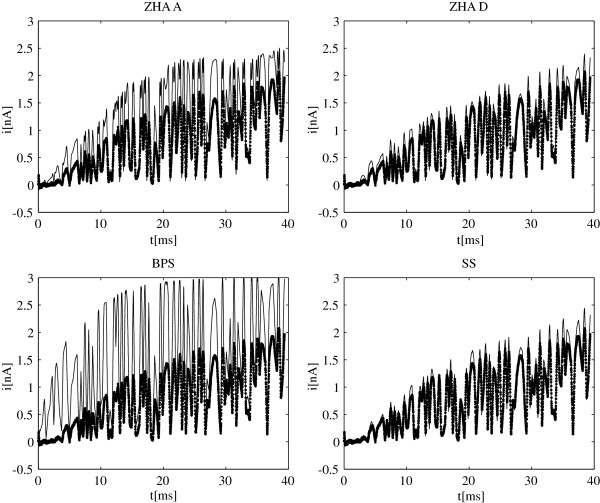
**Model currents for wavelet-based voltage pulses.** Responses of the four models to voltage pulses from Figure 3 A) are shown. Model currents (solid line) are compared to the experimental ionic currents (dotted line). Data is shown for models: ZHA A, ZHA D, BPS, and SS.

**Figure 5:**
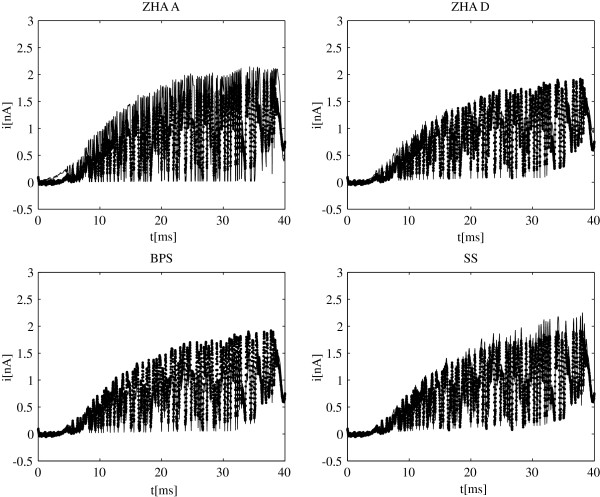
**Model currents for wavelet-based voltage pulses.** Responses of the four models to voltage pulses from Figure 3C) are shown. Model currents (solid line) are compared to the experimental ionic currents (dotted line). Data is shown for models: ZHA A, ZHA D, BPS, and SS.

Comparing model currents and ionic currents from electrophysiological experiments is the ultimate test for any model of channel gating. In most studies the fit is limited to the activation and tail ionic currents. Sometimes other voltage protocols are added but they typically consist of few voltage steps at discrete times. As noted earlier (see Figure 2) with the exception of ZHA A all models seem to adequately fit the standard sets of data for stepped-voltage protocols. We also computed responses of models ZHA A, ZHA D, BPS and SS to the wavelet-based voltage pulses using equation (14). In Figure 4, these model currents are plotted against the experimental ionic currents recorded for these same voltages waveforms. As expected the ZHA A model does a poor job approximating the experimental data. The new result is the BPS model which fit the activation and tail data very well but fails to fit the wavelet-based protocols. It suggests that the model has deficiencies and should be discarded in favor of models ZHA D or SS. As for the latter two this set of protocols still does not distinguish well between them, however, the algorithm can be used again to develop new set of pulses that would maximize differences in responses of these two remaining models. These protocols would then need to be implemented in another patch-clamping experiment and we one of them would prove superior to the other. Figure 5 shows the responses to a higher frequency voltage pulse constructed using 10 wavelet levels. While it still does not entirely distinguish between models ZHA D and SS, it shows that the former overestimates a little the rate of activation while the latter seems to underestimate steady-state currents.

## Discussion

The goal of this paper is to describe a new method for developing voltage protocols for ion channel patch-clamping experiments. It is expected that in order to obtain new information about channel gating kinetics or to explore new phenomena that ion channels may exhibit, one must go beyond the typical set of stepped voltage protocols. The method we develop allows tailoring the voltage inputs in a patch-clamping experiment to maximize a desired output. The design is based on the inverse discrete wavelet transform where the voltage pulse is constructed as a superposition of wavelets in a dyadic wavelet basis. Each such pulse is characterized by the corresponding set of wavelet coefficients and it can be synthesized using a variety of available numerical packages. The desired outcome of the patch-clamping experiment needs to be quantified through a suitably defined cost functional and then the design algorithm is essentially an optimization procedure where the set of wavelet coefficients is optimized to maximize (or minimize) the cost functional. The optimization procedure we used is a search of the parameter space through a random genetic algorithm. We used a method where the “daughter” parameter sets are generated by randomly perturbing the parameters of the “parent” set, with a range of random fluctuations decreasing through the generations. This ensures convergence but also avoids trapping in a local extremum of the cost functional. We used this method previously e.g. for fitting of kinetic models of channel gating. Other algorithms are possible, for instance [32] mentions generating daughter sets by an algebraic formula that combines randomly generated intermediate sets and the “parent” set. Also, in our application we used the Daubechies 8 wavelets, however other types, as well as wavelet packet could be used. An interesting case would be using the Haar wavelets as they would be a natural extension of the voltage-step protocols currently dominating ion channel experimental practice.

This method has many potential uses but we apply it to a specific example: testing of kinetic models of ion channel gating. It is known that the models are notoriously ambiguous. Different research groups propose very different models that match available data equally well, hence there is no way to decide which model (or maybe none) is the best approximation of reality. It is accepted that in order to improve the model design and testing process, we need to include more data and that new data should be substantially different from all existing data. Just adding more of the same will not improve the process. There are two approaches to this. One is to get new data that is of different physical nature. The best illustration would be to add gating currents to previously used ionic currents, or to use single channel data in addition to whole-cell data. This is not the focus of this manuscript. We consider the second approach where the new data is of the same physical nature as previously (i.e. the same physical quantity is measured) but the input to the system has been substantially altered. In recent years there has been interest in using fluctuating voltages as opposed to piecewise constant voltages [10-17,29,30], as well as using conductance hysteresis curves to differentiate between different models [39,40]. The rationale is that this new type of stimulus causes a nonequilibrium response of the ion channel ensemble and some models that were built based on equilibrium (or near-equilibrium) experimental data will fail to reproduce the nonequilibrium data. The main issue with this approach is to decide what type of fluctuating voltage will work best. In the previous studies on this method (named the Nonequilibrium Response Spectroscopy) [10,11,17] the dichotomous noise was used but there was no systematic way to determine the properties of the noise that would allow the most efficient model evaluation. In fact, the choices were fairly arbitrary and amounted to scanning a range of noise amplitudes and frequencies and hoping to find the “right” range. The essential parameters are the range of fluctuations and the spectro-temporal properties, i.e. what frequencies are present in the signal and at what times (the signal need not be stationary). The method we described allows the selection of these properties and is essentially an optimization procedure. We have selected four models proposed by leading electrophysiology labs and defined the cost functional as the difference between the model currents generated by these models, measured as the χ^2^ error. Then we constructed several pulses that maximize that cost functional, i.e. pulses which when input into these models generate maximally divergent outputs. These model currents were then compared to the experimental currents recorded in patch-clamping experiments, in response to the same voltage inputs. This facilitated the selection of the best models, and the process can be done iteratively. A preliminary version of this method was previously applied to models of a human heart sodium channel [16]. This is, however, only one possible application of this method. Another putative application would be to explore the possibility of kinetic focusing of ion channels. This term refers to a selective enhancement of a particular state of ion channels in response to a fluctuating stimulus in terms of the applied voltage. In the first paper describing the phenomenon [29] it was suggested that it could be generated by a dichotomous noise voltage and a preliminary experimental study [30] also used that same type of voltage stimulus. The method we describe here would make it possible to design rapidly fluctuating voltages that would maximize the occupancy of a particular conformational state of the ion channels. Further theoretical and experimental studies are in progress [31]. Finally, we mention that a related method has been proposed in engineering for optimizing outputs of nonlinear systems subject to energy constrains, and illustrated with examples of an aircraft model and of a bistable potential system [32].

## Conclusions

In this article we describe a new method that represents a radical departure from the current paradigm in ion channel electrophysiology. We propose using fluctuating voltages through a patch-clamp apparatus. While the use of fluctuating inputs to nonlinear systems to elicit new types of system responses is well known and researched in different areas of physics, our method has the added advantage of allowing tailoring inputs to achieve a particular, most desired outcome. If we quantify the “desirability” of outcome through a suitable “cost functional”, through our method one can construct an input that optimizes this cost functional. The pulse design is based on the wavelet decomposition. In particular, we implement the method in ion channel electrophysiology where the input is the voltage applied in patch-clamp experiments, the output – the measured whole-cell ionic current and the “cost functional” – the fit of various Markov models to the experimental data. Our method can be used to aids selection and testing of models of channel gating kinetics. We also comment on other possible applications of this method to electrophysiology and other fields.

## Competing interests

The author declares that he no competing interests exist.

## Authors’ contributions

AK performed the all experimental and numerical work presented in this paper and designed the method presented.

## Supplementary Material

Additional file 1 A function file which accepts the model parameters of the SS model and the applied voltage as its inputs and returns the transition matrix for this model. Click here for file

Additional file 2 A function file which accepts the model parameters of the ZHA D model and the applied voltage as its inputs and returns the transition matrix for this model. Click here for file

Additional file 3: **A function file that accepts two discrete time series as its arguments and returns the χ**^**2**^** error between the series, normalized to the number of sampling points.** Click here for file

Additional file 4: **Matlab code to optimize a pulse synthesized from wavelets.** The program uses the difference between the computed responses of models SS and ZHA D (computed as χ^2^ error) as the cost functional and optimizes the pulse for maximal value of the cost functional. Click here for file

## References

[B1] Hille B Ionic Channels of Excitable Membranes 1992 Sunderland (MA): Sinauer Associates Inc.

[B2] Stefani E Toro L Perozo W Bezanilla F Gating of Shaker K^+^ channels: I. Ionic and gating currents Biophys J 1994 66 996 1010 10.1016/S0006-3495(94)80881-1 8038403 PMC1275807

[B3] Bezanilla F Peroso E Stefani E Gating of Shaker K + channels II. The components of gating currents and a model of channel activation Biophys J 1994 66 1011 1021 10.1016/S0006-3495(94)80882-3 8038375 PMC1275808

[B4] Schoppa NE Sigworth FJ Activation of Shaker potassium channels I. Characterization of voltage-dependent transitions J Gen Physiol 1998 111 271 294 10.1085/jgp.111.2.271 9450944 PMC2222764

[B5] Schoppa NE Sigworth FJ Activation of Shaker potassium channels II. Kinetics of the V2 mutant channel J Gen Physiol 1998 111 295 311 10.1085/jgp.111.2.295 9450945 PMC2222768

[B6] Schoppa NE Sigworth FJ Activation of Shaker potassium channels III. An activation gating model for wild-type and V2 mutant channels J Gen Physiol 1998 111 313 342 10.1085/jgp.111.2.313 9450946 PMC2222769

[B7] Hoshi T Zagotta WN Aldrich R Shaker potassium channel gating I. Transitions near the open state J Gen Physiol 1994 103 249 278 10.1085/jgp.103.2.249 8189206 PMC2216835

[B8] Zagotta WN Hoshi T Dittman J Aldrich R Shaker potassium channel gating II. Transitions in the activation pathway J Gen Physiol 1994 103 279 319 10.1085/jgp.103.2.279 8189207 PMC2216838

[B9] Zagotta WN Hoshi T Aldrich R Shaker potassium channel gating III. Evaluation of kinetic models for activation J Gen Physiol 1994 103 312 362 10.1085/jgp.103.2.321 PMC2216839 8189208

[B10] Sakmann B Neher E Single-channel recording 1995 New York, London: Plenum Press

[B11] Millonas MM Hanck DA Non-equilibrium response spectroscopy of voltage-sensitive ion channel gating Biophys J 1998 74 210 229 10.1016/S0006-3495(98)77781-1 9449324 PMC1299376

[B12] Millonas MM Hanck DA Non-equilibrium response spectroscopy and the molecular kinetics of proteins Phys Rev Lett 1998 80 401 404 10.1103/PhysRevLett.80.401

[B13] Kargol A Application of the ensemble nonequilibrium response spectroscopy to Shaker potassium ion channel gating Cell Mol Biol Lett 2004 9 2 375 388 15213816

[B14] Kargol A Hosein-Sooklal A Optimal sensitivity analysis of ion channel gating kinetics J Membrane Biol 2004 199 113 118 10.1007/s00232-004-0681-z 15383921

[B15] Kargol A Hosein-Sooklal A Constantin L Przestalski M Application of oscillating potentials to Shaker potassium channel Gen Physiol Biophys 2004 23 53 75 15270129

[B16] Hosein-Sooklal A Kargol A Wavelet analysis of nonequilibrium ionic currents in human heart sodium channel (hH1a) J Membrane Biol 2002 188 199 212 10.1007/s00232-001-0188-9 12181611

[B17] Kargol A Smith B Millonas MM Application of nonequilibirum response spectroscopy to the study of channel gating. Experimental design and optimization J Theoret Biol 2002 218 239 258 10.1006/jtbi.2002.3073 12381295

[B18] Hanggi P Stochastic resonance in biology Chem Phys Chem 2002 3 285 290 10.1002/1439-7641(20020315)3:3<285::AID-CPHC285>3.0.CO;2-A 12503175

[B19] Adair RK Noise and stochastic resonance in voltage-gated ion channels PNAS 2003 100 12099 12104 10.1073/pnas.2034447100 14506291 PMC218719

[B20] Wiesenfeld K Jaramillo F Minireview of stochastic resonance Chaos 2001 8 539 548 12779757 10.1063/1.166335

[B21] Goychuk I Hanggi P Vega JL Miret-Artes S Non-Markovian stochastic resonance: Three-state model of ion channel gating Phys Rev E 2005 71 061906 10.1103/PhysRevE.71.061906 16089764

[B22] Agudov NV Krichigin AV Valenti D Spagnolo B Stochastic resonance in a trapping overdamped monostable system Phys Rev E 2010 81 051123 10.1103/PhysRevE.81.051123 20866201

[B23] Guo F Effect of White Noise and Dichotomous Noise in a Bistable System Adv Mat Res 2011 2143 295 297

[B24] Miyamoto S Nishiguchi K Ono Y Itoh KM Fujiwara A Resonant escape over an oscillating barrier in a single-electron ratchet Phys Rev B 2010 82 033303

[B25] Orlandia JG Sancho JM Theoretical study of a membrane channel gated by ATP Eur Phys J E 2009 29 329 336 10.1140/epje/i2009-10483-9 19575251

[B26] Lee K Sung W Effects of nonequilibrium fluctuation on ionic transport through biomembranes Phys Rev E 1999 60 4681 4686 10.1103/PhysRevE.60.4681 11970332

[B27] Flomenbom O Klafter J Resonant activation in discrete systems Phys Rev E 2004 69 051109 10.1103/PhysRevE.69.051109 15244810

[B28] Li J Han Y Escape over fluctuating potential barrier for system only driven by dichotomous noise Phys Lett A 2006 359 573 576 10.1016/j.physleta.2006.07.055

[B29] Millonas MM Chialvo D Control of Voltage-Dependent Biomolecules via Nonequilibrium Kinetic Focusing Phys Rev Lett 1996 76 550 553 10.1103/PhysRevLett.76.550 10061485

[B30] Kargol A Kabza K Test of nonequilibrium kinetic focusing of voltage-gated ion channels Phys Biol 2008 5 026003 10.1088/1478-3975/5/2/026003 18497445

[B31] Ponzoni L Celardo GL Borgonovi F Kaplan L Kargol A Focusing in multiwell potentials: applications to ion channels. arXiv:1209.0505 [cond-mat.mes-hall] 2012 10.1103/PhysRevE.87.052137 23767517

[B32] Watson GH Gilholm K Jones JG A wavelet-based method for finding inputs of given energy which maximize the outputs of nonlinear systems Int J Syst Sci 1999 30 1297 1307 10.1080/002077299291598

[B33] Kramers HA Brownian motion in a field of force and the diffusion model of chemical reactions Physica 1940 7 284 304 10.1016/S0031-8914(40)90098-2

[B34] Sigg D Qian H Bezanilla F Kramer’s diffusion theory applied to gating kinetics of voltage-dependent ion channels Biophys J 1999 76 782 803 10.1016/S0006-3495(99)77243-7 9929481 PMC1300081

[B35] Sigg D Bezanilla F A physical model of potassium channel activation: from energy landscape to model kinetics Biophys J 2003 84 3703 3716 10.1016/S0006-3495(03)75099-1 12770877 PMC1302953

[B36] Qian H From discrete protein kinetics to continuous Brownian dynamics: A new perspective Protein Sci 2002 11 1 5 10.1110/ps.ps.18902 11742116 PMC2368777

[B37] Daubechies I Ten lectures on wavelets 1992 Philadelphia: SIAM

[B38] Wallisch P Matlab for neuroscientists 2009 Amsterdam: Academic Press

[B39] Andersson T Exploring voltage-dependent channels *in silico* by hysteretic conductance Math Biosci 2010 226 16 27 10.1016/j.mbs.2010.03.004 20303991

[B40] Das B Banerjee K Gangopadhyay G Entropy hysteresis and nonequilibrium thermodynamic efficiency of ion conduction in a voltage-gated potassium ion channel Phys Rev E 2012 86 061915 10.1103/PhysRevE.86.061915 23367983

